# The missing link – the role of primary care in global health

**DOI:** 10.3402/gha.v7.23693

**Published:** 2014-02-13

**Authors:** Mala Rao, Eva Pilot

**Affiliations:** 1 Institute for Health and Human Development, University of East London, London, UK; 2 Department of International Health, CAPHRI School for Public Health and Primary Care, Faculty of Health, Medicine and Life Sciences, Maastricht University, Maastricht, The Netherlands

**Keywords:** primary care, global health, universal health coverage, health equity

## Abstract

This chapter provides an overview of the role of primary care in the context of global health. Universal health coverage is a key priority for WHO and its member states, and provision of accessible and safe primary care is recognised as essential to meet this important international policy goal. Nevertheless, more than three decades after Alma Ata, the provision of primary health care remains inadequate, indicating that primary care has not received the priority it deserves, in many parts of the world. This is despite the proven health benefits that result from access to comprehensive primary health care. We highlight some examples of good practice and discuss the relevance of primary care in the context of health equity and cost-effectiveness. Challenges that influence the success of primary care include the availability of a qualified workforce, financing and system design and quality assurance and patient safety.

*Pregnant women waiting hours for a routine antenatal check in a frantically busy and dirty out-patients department of a large maternity hospital. An infant dying due to severe diarrhoea by the time its parents reach the nearest health care facility, a hospital many kilometers away.*

*A middle-aged woman having to guess whether it would be best to consult a gynaecologist or a gastroenterologist for her stomach ache.*

*An overweight smoker discovering that he is diabetic only after he has suffered his first heart attack*.

Scenarios which remain all too familiar in many parts of the world today.

Would these cases arise if good quality dependable primary care was a routinely available part of the national system for delivering health care?

## Empty promises

In 1978, the WHO Alma Ata agreement between 134 countries acknowledged ‘health as a foremost human right’ and identified primary healthcare as pivotal to delivering health to all by 2000. It was envisaged as ‘the first level of contact of individuals, the family and community with the national health system bringing health care as close as possible to where people live and work, and constituting the first element of a continuing health care process’ (1). Furthermore, it was expected to address the main health problems in the community, through promotive, preventive, curative and rehabilitative care.

But time has shown that this vision remains unfulfilled in much of the world. Many developing countries strayed from this original promise in preference for vertical health care programmes focusing on individual priorities such as malaria and tuberculosis. Whilst some of these programmes have been successful in controlling specific diseases, they have resulted in a fragmentation of care and an exponential growth in hospitals offering specialist and super-specialist treatments in response to other health demands (2).

Primary care remains overlooked in parts of the developed world also, with the US providing a striking example of the imbalance between primary and specialist care (3). This has been driven by the availability of greatly expanded specialist diagnostic and therapeutic options due to the growth of medical technologies, as well as insurance-based health financing which has not only freed patients of any financial constraints to seeking specialist care but also encouraged hospital-based specialized services as a result of payment policies which favour complex care in preference to preventive services or routine consultations.

Comprehensive research evidence is now available to demonstrate that this hospital-oriented health delivery landscape is unsuitable to delivering the vision of Alma Ata. Nevertheless, primary care remains undervalued in many parts of the world where the professional status and remuneration of primary care staff may be low, policy makers have little appreciation or knowledge of the contribution of primary care to high-quality health systems and the public has scant regard for the services offered at that level. The objective of this paper is to summarize the evidence which highlights why primary care is of crucial importance to improving population health and the challenges as well as opportunities to achieving better primary care.

### What is primary care?

Barbara Starfield described primary care as ‘the provision of first contact, person-focused, ongoing care over time that meets the health-related needs of people, referring (to hospital) only those problems too uncommon to maintain competence and coordinates care when people receive services at other levels of care’ (4). In its 2008 World Health Report entitled *Primary Care Now More Than Ever* (2), the World Health Organization extended the definition to include involvement of the ‘citizen–patient’ and the community in primary care (5). It also reiterated the Alma Ata declaration's vision of intersectoral collaboration, social justice and equity, with actions to address the social determinants of health as a key constituent of a primary care strategy equipped to achieve these aspirations (6, 7).

### The health benefits of primary care

Starfield published a mass of evidence to show that a health system based on strong primary care delivers better population health outcomes at lower cost (4, 8, 9) and can counteract the adverse impacts of poor economic conditions on health (3). This has been consistently demonstrated in low- and middle-income countries and in countries of the Organization of Economic Development (OECD) (5), as well as by different types of studies, international comparisons, population studies within countries and clinical studies (10).

The example of cardiovascular disease is illustrative of how human and economic costs of ill health may be reduced, if a high-quality primary care service is in place to offer preventive care or early detection and management in the early stages. The natural history of cardiovascular disease starts long before symptoms manifest themselves. Relatively less expensive preventive measures and early diagnosis and management options available within the primary care setting, for instance, to patients who are obese, or suffer from diabetes or hypertension, have the benefit of preventing premature death and reducing the burden of cardiovascular illness of increasing severity, as well as the costs of expensive secondary and tertiary care. The cost-effective management of many conditions is dependent on strong primary care services offering preventive and promotive care, as well as treatment for chronic diseases, which can be provided in a primary care setting.

Primary care oriented countries have fewer low birth weight infants, lower infant mortality, especially post-neonatal mortality, fewer years of life lost due to suicide, fewer years of life lost due to all except external causes and higher life expectancy at all ages except at 80 years (10, 11). Greater availability of primary care has also been shown to increase satisfaction with the health care system and decrease utilization of hospital care and emergency department visits (3). While the strongest evidence of benefit has been reported from the OECD countries where the most rigorously designed studies have been conducted, similar evidence is also emerging from other parts of the world. A recently published study of 102 low- and middle-income member states of the WHO using the 2011 World Health Statistics demonstrated that greater availability of primary health care was significantly associated with longer life expectancy and lower infant mortality and under-five mortality. This contrasted with the negative health outcomes found to be related to increased health expenditure as a share of the country's GDP, confirming that good health could be achieved at low cost (12–14).

### Primary care and health equity

The poor have less financial, geographic and cultural access than the rich to good quality hospital services even in urban areas. Primary care has the advantage of greater accessibility to the community and across the social gradient. Where primary care services are lacking, hospitals find themselves overwhelmed with patients with minor and major health problems jostling for attention in environments more suited to the care of life-threatening illness, thus wasting scarce specialist resources. By contrast, evidence gathered by the World Bank has highlighted that primary care is capable of managing 90% of health care demands, with only the remaining 10% requiring services associated with hospitals (15).

Research into the benefits of primary care has also demonstrated that ‘the availability of primary care services improves patients’ self-perceived health status. Furthermore, the longitudinal care afforded by primary care services is independently associated with improved patient satisfaction, reduced use of ancillary and laboratory tests, improved patient compliance, shorter length of stay in a hospital, and improved recognition of patients’ behavioural problems (1). Importantly, primary care offers access to preventive, promotive and curative care, which may explain a key finding of experts that countries and areas within countries with strong primary care generally have healthier populations.

Of the eight UN Millennium Development Goals, three are directly health-related: to reduce infant mortality by two-thirds by 2015, to reduce maternal mortality by 75% and to tackle HIV/AIDS, malaria and TB. In India, for example, although there is optimism that the goal to reduce HIV/AIDS, malaria and TB may be met, there is great concern that the infant and maternal mortality targets may be missed. According to the Planning Commission of India, a severe shortfall in primary care services may be an important underlying factor, undermining access to immunizations, antenatal care, nutritional advice and early diagnoses of simple-to-treat but potentially life-threatening conditions such as diarrhoea in children (16).

There is also evidence that primary care improves the management of chronic illnesses that have serious consequences if neglected. In 2011, the UN summit on non-communicable diseases (NCDs) alerted member states to their enormous burden of diseases such as cancer, diabetes and hypertension, and to the fact that these have replaced infectious diseases as the major cause of death. NCDs have an impact not just on the family, but on the economy too, with studies demonstrating a clear link between rising levels of NCDs and a loss of economic growth. High-quality primary care has been shown to lower mortality linked to medical problems such as these by focusing on prevention, and early diagnosis and treatment (17).

## The success of primary care: some examples

Many countries have successfully adopted a primary-care-centred health care system and accrued substantial health benefits as a result.

One great exponent of primary health care is Cuba. Despite overwhelming economic difficulties, its health care thrives in a model that is both sustainable and effective. Its success lies in the focus on early intervention rather than on end-stage disease treatment. With an average life expectancy of 78 years, child mortality which has fallen from 53/1,000 to 7/1,000 in the last 40 years, and the patient education and health promotion offered to its population of 11.3 million people, the amount of medical supplies required and the burden on secondary and tertiary health care have dramatically reduced. In 2004, Cuba spent only 7% of its GDP on health care compared to 10% by the UK, and 13% by the USA – equating to £7 per capita spent in Cuba in comparison to a 400 hundred times greater £2,870 per capita in the USA (18–22).

The lower morbidity and mortality rates in the Indian state of Kerala are attributed to the strong primary care base on which the health system is built. Researchers have attributed the reduced costs of patient care to an increase in the number of primary health centres that have lowered the burden on secondary and tertiary care by differentiating and treating the minor ailments compared to the major ones, though over 50% of health care in Kerala is still private. Over 80% of infants receive vaccines within the first year of life and government funding of primary care is reported to have made it more accessible and available to all, providing an example for other states to follow (23, 24).

Sri Lanka has also proved that primary care is a useful cog in the machine of public health for the country. Consequently, the life expectancy stands at 73 years, with infant and maternal mortality rates reduced to an impressive 16/1,000 and 30/1,000 respectively. A notable example from Africa is Ethiopia (25), where health care has recently been re-organized so as to improve population coverage and eliminate problems such as long-distance travelling to reach health care facilities – two challenges which rural populations in many other countries face daily. Chile, where a recent evaluation demonstrated that primary care clinics which followed a family health care model were more effective than traditional health centres on technical indicators and user ratings (26), is a further exemplar.

## The cost-effectiveness of primary care

A study published in 2011 comparing the cost-effectiveness of the US, the UK and 17 other Western countries concluded that in terms of economic input versus clinical output, the USA health care system was one of the least cost-effective in reducing mortality rates, whereas the UK's national health service was one of the most cost-effective over the period (27). Whilst this study did not explore the reasons for this difference and many factors may have influenced the differences, it did highlight that in the UK, frontline staff had achieved more for less. The UK's health care system with its strong primary care base may have contributed to this finding, at least in part. Statistics from the Royal College of General Practitioners highlight that 90% of health care in the UK is carried out at the primary care level, with the majority of residents registered with a GP and approximately 300 million consultations taking place in a year. Yet, only 24% of the UK health care budget in 2005 was spent on primary care in comparison to the 57% spent on secondary care (28).

More recently, Haggerty and Lévesque (29) reported the findings of a study carried out by Kringos and colleagues (30) across 31 European countries which demonstrated that population health was better in countries which had a strong primary care structure as measured by the density of primary care providers and the quality of their work environment. Better coordination and comprehensiveness of primary care were associated with lower rates of avoidable admissions and fewer potential years of life lost, and countries with higher levels of patient satisfaction with interpersonal dimensions of care also had greater equality of self-rated health. But stronger primary care structures were also associated with higher levels of health care spending after adjustments for GDP per capita, although the rate of growth in health care spending was lowered. The conclusion was that investing in high-quality primary care may not save money in the short-term but results in better population health outcomes and slows the rise in health care costs.

### Challenges to primary care

There are a number of barriers to establishing high-quality primary care and ensuring equity of access across the population. Some methods of health care financing may militate against universal access to primary care. The introduction of user fees has been consistently shown to reduce service utilization. Although tax-based health care financing is most likely to achieve equity of access to services (31, 32), it may result in low levels of funding available for health care, leading to under-investment in primary care and the persistence of poor quality systems (33). Consequently, publicly funded primary care services are perceived as being of low quality, and compel poor families to pay out-of-pocket to consult private doctors. Such a scenario is more prevalent in lower income countries. By contrast, many richer countries have used general revenue funded health systems to reduce the discontinuity of care experienced by patients if out-of-pocket payments are required to be made at each point of contact, and by introducing into primary care a strong gatekeeping function to restrict referrals to the more expensive secondary care (34). The tradition of donor agencies to finance the delivery of targeted health services rather than encourage the reorientation of health systems to establish strong primary care reduces the opportunities for low-income countries to adopt best practice from the richer countries.

Studies have demonstrated that expenditure on medicine accounts for the largest component of out-of-pocket expenditure in both public and private facilities. An analysis of the 2003 World Health Survey data collected from 39 low- and middle-income countries showed that on average, medicines represented over 57% of outpatient out-of-pocket expenditure at public facilities and over 45% of outpatient out-of-pocket expenditure at private facilities (35). Consultation fees were the second largest component, representing on average, 22% of out-of-pocket expenditure at public facilities and 40% of out-of-pocket expenditure at private facilities (35). Recent research indicates that broadened health coverage with extended risk pooling and prepayments rather than out-of-pocket payments leads to better access to necessary care and improved population health, with the largest gains for the poorer section of society (36).

NCDs such as heart disease, diabetes and stroke are the leading causes of death and disability in both the developed and the developing world (37). There is now a vast body of evidence which shows that common modifiable risk factors underlie the prominent diseases, but that these are amenable to treatment as well as support for behaviour change which can be provided in a primary care setting and can vastly improve life expectancy and quality of life if provided on a regular basis. However, in many countries, a diagnosis of NCD may result in a lifelong burden of out-of-pocket expenditure for medicines which poor households can ill afford and is an important cause of poor compliance with treatment. For primary care delivery to be effective, access to affordable or free medicines is essential.

Human resources are among the most important components of a health system's inputs (38). The performance of a primary health care centre depends ultimately on adequate staffing levels and on the knowledge, skills and motivation of the team responsible for delivering services. While ‘one size will not fit all’, evidence from countries with strong primary-care-based health systems such as the UK and the Netherlands has demonstrated how comprehensive primary care can be provided by a multi-disciplinary team with staff ranging from community health workers to nurses and doctors. Expenditure on human resources is usually the biggest single item in the recurrent budget for health (38).

### Patient safety in primary care

The success of primary care also depends on the quality of system design and financing (36). Implementation strategies such as good governance, maintaining of quality standards and targeting vulnerable groups are critical elements. Unfortunately quality assurance and patient safety issues in primary care had received little attention till very recently. Serious gaps in data and knowledge exist for many regions, particularly for developing and transitional countries (39). In early 2012, a Safer Primary Care Expert Working Group with experts from several WHO member states representing all of the six world regions was established to identify and review evidence and to highlight knowledge gaps and key areas for actions. A number of critical patient safety issues in primary care were identified by this Group through a systematic literature review. The Group's recommendations published in the WHO report Safer Primary Care – A Global Challenge call for: ‘both better understanding of the epidemiology of unsafe care, including the causality of adverse events and patient harm, and development of new solutions to improving safety’ in primary care (40). Concerted action will be needed to further develop this into a global roadmap to safer primary care.

## Conclusion

In conclusion, primary care offers much more than simple reduction of costs of a country's health. Experts in the field of primary care research have summarized a number of mechanisms by which intervention at the primary care level can benefit the population: these include increasing accessibility of health to deprived populations, improving overall long term patient care and health, preventative and educational measures (e.g. smoking cessation, early treatment of diabetes), appropriate and focused direction of care (i.e. correct specialist referral) and a reduction in unnecessary, inappropriate medical care. It also helps to narrow the gap between socially deprived and socially advantaged populations.

The continuity and doctor–patient relationships offered by family oriented primary care, alongside the patient education, early intervention and treatment, chronic disease management, counselling and reassurance offered to patients would be impossible to provide in a secondary care setting.

Against a background of the recent global economic downturn, massive demographic shifts and increasing health impacts of climate change adding to the health challenges facing humanity, it is abundantly clear that all countries will need to invest in a primary-care-centred health delivery system, if universal access to health care is to be realised (41). In 2008, Greenhalgh described the dramatic epidemiological change which has taken place in Alma Ata, now renamed Almaty, where the burden of disease has shifted from a ‘third-world’ pattern to a ‘transition country’ pattern, with high levels of obesity and diseases associated with smoking, alcohol and drugs, as well as accidents and violence (42). Irrespective of where a town or country is, within that epidemiological spectrum, high-quality primary care is the only way it will achieve good health for all.
